# Patellariopsidaceae Fam. Nov. With Sexual-Asexual Connection and a New Host Record for *Cheirospora botryospora* (Vibrisseaceae, Ascomycota)

**DOI:** 10.3389/fmicb.2020.00906

**Published:** 2020-05-26

**Authors:** Anuruddha Karunarathna, Derek Peršoh, Anusha H. Ekanayaka, Ruvishika S. Jayawardena, K. W. Thilini Chethana, Ishani D. Goonasekara, Ratchadawan Cheewangkoon, Erio Camporesi, Kevin D. Hyde, Saisamorn Lumyong, Samantha C. Karunarathna

**Affiliations:** ^1^Department of Biology, Faculty of Science, Chiang Mai University, Chiang Mai, Thailand; ^2^Department of Entomology and Plant Pathology, Faculty of Agriculture, Chiang Mai University, Chiang Mai, Thailand; ^3^World Agroforestry Centre, East and Central Asia, Kunming, China; ^4^Key Laboratory for Plant Diversity and Biogeography of East Asia, Kunming Institute of Botany, Chinese Academy of Sciences, Kunming, China; ^5^Centre of Excellence in Fungal Research, Mae Fah Luang University, Chiang Rai, Thailand; ^6^AG Geobotany, Ruhr-University Bochum, Bochum, Germany; ^7^Research Center of Microbial Diversity and Sustainable Utilization, Faculty of Science, Chiang Mai University, Chiang Mai, Thailand; ^8^A.M.B, Circolo Micologico “Giovanni Carini”, Brescia, Italy; ^9^A.M.B. Gruppo, Micologico Forlivese “Antonio Cicognani”, Forlì, Italy; ^10^Academy of Science, The Royal Society of Thailand, Bangkok, Thailand

**Keywords:** Ascomycetes, *Cheirospora botryospora*, Leotiomycetes, Pezizomycotina, sporodochium

## Abstract

Helotiales is a polyphyletic order of Ascomycetes. The paucity of relevant molecular data and unclear connections of sexual and asexual morphs present challenges in resolving taxa within this order. In the present study, Patellariopsidaceae fam. nov., the asexual morph of *Patellariopsis atrovinosa*, and a new record of *Cheirospora botryospora* (Vibrisseaceae) on *Fagus sylvatica* (Fagaceae) from Italy are discussed based on morphology and molecular phylogeny. Phylogenetic analyses based on a combined sequence dataset of LSU and ITS were used to infer the phylogenetic relationships within the Helotiales. The results of this research provide a solid base to the taxonomy and phylogeny of Helotiales.

## Introduction

The Leotiomycetes (Pezizomycotina) is a very diverse class and was erected when the super-class Leotiomyceta was split in to seven classes by Eriksson & Winka (Eriksson and Winka, 1997). Leotiomycetes currently comprises 13 orders, out of which eight are monotypic, while over 200 genera are represented by one species only (Baral, 2016; Wijayawardene et al., 2018; Ekanayaka et al., 2019; Johnston et al., 2019). Among the orders in Leotiomycetes, Helotiales consists of the highest number of genera, *incertae sedis* within the familial rank (ca. 90–151) (Baral, 2016; Quijada et al., 2018; Wijayawardene et al., 2018). Hawksworth (2001) estimated that Helotiales consists of 70,000 species. Only 2,334 species belonging to 423 genera in 25 families have been recorded in Helotiales. This constitutes half of all known species in Leotiomycetes (Ekanayaka et al., 2019).

Recent phylogenetic studies based on ribosomal DNA analyses have reported the polyphyletic nature of Helotiales (Ekanayaka et al., 2019; Johnston et al., 2019). The lack of knowledge between asexual and sexual morph connections complicates the systematics of this order (Wang et al., 2006b). Many helotialean fungi are known based on a sexual morph, with their asexual morphs being either undiscovered or assumed to have been lost in evolution (Wang et al., 2006b). On the other hand, it is suggested that asexual morphs from various environmental samples are members of Helotiales, with no mention of their sexual morphs (Sutton and Hennebert, 1994; Marvanova et al., 1997).

Helotiales is the largest group of non-lichen forming ascomycetes and occur in a wide range of niches (Ekanayaka et al., 2017; Wijayawardene et al., 2017). The members of Helotiales are recorded as plant pathogens, endophytes, nematode-trapping fungi, mycorrhizae, fungal parasites, terrestrial and aquatic saprobes, root symbionts and wood rot fungi (Wang et al., 2006a).

The objectives of this study are to introduce a new family with their sexual-asexual inter-connection and to provide a new host record for *Cheirospora* in Vibrisseaceae.

## Materials and Methods

### Plant Sample Collection, Morphological Studies and Isolation of Pure Culture

Dead aerial branches of *Fagus sylvatica* L. (Fagaceae) and *Corylus avellana* L. (Betulaceae) were collected from Passo la Calla, Stia (province of Arezzo [AR]) Italy and Fiumicello di Premilcuore (province of Forlì-Cesena [FC]) Italy, respectively. Specimens were preserved and observed following the method of Karunarathna et al. (2017). Hand-cut sections of the fruiting structures were mounted in water for microscopic studies and photomicrography. Specimens were examined with a Nikon ECLIPSE 80i compound microscope and photographed with a Canon EOS 600D digital camera fitted to the microscope. Measurements of morphological characteristics were made with the Tarosoft (R) Image Frame Work program and images used for figures were processed with Adobe Photoshop CS3 Extended version 10.0 (Adobe Systems, United States).

Single spore isolation was carried out following the method described in Chomnunti et al. (2014). Germinated spores were individually transferred to potato dextrose agar (PDA) plates and grown at 10–16°C. Colony color and other characteristics were observed and measured after 1 week and 3 weeks. The specimens were deposited in the Mae Fah Luang University Herbarium (MFLU), Chiang Rai, Thailand. Living cultures were deposited in Mae Fah Luang Culture Collection (MFLUCC). Facesoffungi (FoF) and Index Fungorum numbers (IF) were acquired as in Jayasiri et al. (2015) and Index Fungorum (2019).

### DNA Extraction, PCR Amplification, and Sequencing

Genomic DNA was extracted from fresh fungal mycelium grown on PDA media at 16°C for 4 weeks using the Biospin Fungus Genomic DNA Extraction Kit (BioFlux^®^, Hangzhou, China) following the instructions of the manufacturer.

The DNA amplification was performed by polymerase chain reaction (PCR). A partial sequence of the LSU rRNA gene region was amplified using the primer pair LR0R and LR5 (Vilgalys and Hester, 1990). The internal transcribed spacer regions (ITS1, 5.8S, ITS2) were amplified using the primer pair ITS5 and ITS4 (White et al., 1990). PCR was carried out following the protocol of Phookamsak et al. (2014). The quality of PCR products was checked by gel electrophoresis on 1% agarose gels stained with ethidium bromide. The amplified PCR fragments were sent to a commercial sequencing provider (Shanghai Sangon Biological Engineering Technology & Services Co., Shanghai, China). The sequence data acquired were deposited in GenBank (Table 1).

**TABLE 1:** Taxa used in the phylogenetic analyses and their corresponding GenBank numbers (Newly generated sequences are indicated in black bold).

**Species**	**Strain/**	**GenBank**	
	**Voucher No.**	**Accession No.**	
		**ITS**	**LSU**
*Acidomelania panicicola*	61R8	KF874619	KF874622
*Amicodisca* sp.	KUS_F51377	JN086692	JN033389
*Aquapoterium pinicola*	ATCC MYA-4213	NR_111345	NG_056957
*Arachnopeziza aurata*	KUS-F52038	JN033393	JN086696
*Arachnopeziza aurelia*	KUS-F51520	JN033409	JN086712
*Ascocoryne cylichnium*	KUS_F52351	JN086709	JN033406
*Ascocoryne sarcoides*	HKAS 90651	MK591999	MK584973
*Bryoclaviculus campylopi*	PDD:101074	JX393084	
*Bryoglossum gracile*	DAOM178087	AY789285	
*Bulgariella pulla*	DHP 15-215	KU845540	KU845536
*Cadophora fastigiata*	CBS 869.69	MH859469	MH871247
*Cadophora malorum*	A163	AY249057	AY249080
*Cheirospora botryospora*	CPC 24607	KR611872	KR611894
***Cheirospora botryospora***	**MFLUCC 17-1399**	**MN535816**	**MN535856**
*Chlorosplenium chlora*	BHI_F737a		MG553994
*Chlorosplenium chlora*	BHI_F736a	MG553993	MG553993
*Crucellisporium umtamvunae*	CBS 125742	MH863659	MH875124
*Dicephalospora huangshanica*	MFLU 18-1828	MK591979	MK584979
*Discinella boudieri*	HB4326	KC412001	
*Drepanopeziza ribis*	CBS 200.36	MH855774	MH867284
*Drepanopeziza salicis*	CBS 405.64	MH858467	MH870102
*Encoeliopsis rhododendri*	CBS 905.69	MH859479	MH871259
*Geniculospora grandis*	CBS 261.84	MH873440	MH861735
*Godronia ribis*	CBS 163.66	MH858761	MH870393
*Graddonia coracina*	ILLS60491	JN012009	JQ256423
*Haplographium delicatum*	CBS 196.73	MH872362	MH860659
*Heterosphaeria linariae*	MFLU 15-2764	MK591955	MK585000
*Heterosphaeria patella*	G.M. 2014-08-04-1	MF196187	
*Hyaloscypha bicolor*	CBS 144009	MH018932	MH018943
*Hyaloscypha vitreola*	CBS 126276	MH863954	MH875413
*Hydrocina chaetocladia*	CCM F-10890	KC834062	KC834031
*Hymenotorrendiella madsenii*	ICMP 15648	KJ606676	AY755336
*Lachnum abnorme*	KUS-F52080	JN033395	JN086698
*Lambertella seditiosa*	WU 32446	KF499362	
*Loramyces juncicola*	CBS 293.52	MH857043	MH868576
*Loramyces macrosporus*	CBS 235.53	MH857170	MH868710
*Mitrulinia ushuaiae*	PDD:105643	KX273438	KX273439
*Mollisia cinerea*	CBS 128349	JF514855	MH876343
*Neopyrenopeziza*	MFLU 16-0599	NR_163783	MK592001
*nigripigmentata*			
*Patellariopsis atrovinosa*	G.M. 2014-06-15-1	KY462814	KY462814
*Patellariopsis atrovinosa*	G.M. 2016-05-04-1	KY970066	KY970066
***Patellariopsis atrovinosa***	**MFLUCC 17-1411**	**MN535817**	**MN535857**
*Patellariopsis dennisii*	CBS 174.66	MH858765	MH870396
*Patellariopsis dennisii*	G.M.2017-09-04.3	MK120898	MK120898
*Peltigeromyces* sp.	HB 6432		KX090803
*Phialocephala scopiformis*	CBS 468.94		NR_119460
*Phialocephala urceolata*	UAMH 10827		NR_111285
*Pulvinata tomentosa*	MFLU 18-1819	MK591965	MK584938
*Rhexocercosporidium carotae*	CBS 418.65	MH858647	MH870289
*Rutstroemia longipes*	TNS F-40097	AB926073	AB926142
*Tetracladium marchalianum*	CBS 266.84	MH861736	MH873441
*Trimmatostroma betulinum*	MFLU 15-2991	MK591956	MK584993
*Trimmatostroma salicis*	MFLU 18-0702		MK584996
*Unguicularia unguiculata*	NK322		HG326612
*Varicosporium delicatum*	CCM F-19494	JQ412864	KC834036
*Vibrissea flavovirens*	MBH39316	AY789427	AY789426
*Vibrissea truncorum*	AFTOL_ID 1322		FJ176874

Sequencing of the ITS region of strain MFLUCC 17-1411 was failed due to an intron, of about 1.4 kb in length, positioned between the binding site of primer ITS5 and the start of the ITS region. To obtain a double-stranded ITS sequence, a piece of sporodochium < 0.5 mm^3^ was removed from the specimen and added to a reaction tube with 5 μl of sterile distilled water (dH_2_O). The soaked specimen was frozen (−20°C) and thawn (+20°C) for five times and 0.5 μl of the solution was used for amplification. Based on initially obtained sequence information, a forward primer (Karu_F01: 5′-CAATGATCAAAGCAGTTGCG-3′) was designed, which has similar properties as the ITS4 primer and binds to the intron sequences close its 3′-end. The PCR reaction included 0.5 μl of the DNA-containing solution, 0.25 μl of each primer (Karu_F01 and ITS4; 10 μM, each), 5.25 μl of sterile dH2O and 6.25 μl of the GoTaq^®^ G2 Hot Start Colorless Master Mix (PROMEGA; GoTaq^®^ Hot Start Polymerase in 2 × Colorless GoTaq^®^ Reaction Buffer (pH 8.5), 400 μM dNTPs, 4 mM MgCl2). The PCR commenced with 3 min denaturation at 95°C, followed by 33 amplification cycles (27 s at 94°C, 60 s at 56°C, and 90 s at 72°C) and a final elongation at 72°C for 7 min. The PCR products were cleaned by successive incubation at 37°C for 30 min and 80°C for 15 min after adding 0.2 μl exonuclease I (20.000 U/ml), 0.2 μl Shrimp-Alkaline-Phosphatase (1.000 U/ml; both New England Biolabs) and 1.6 μl sterile dH_2_O to 5 μl of PCR product. Purified PCR products were sequenced by the sequencing service of the Ruhr-Universität Bochum using a Genetic Analyzer 3130xl (Applied Biosystems).

### Phylogenetic Analyses

Phylogenetic analyses were conducted separately based on LSU and ITS gene sequence data. Reference sequences (Table 1) of representative families in Leotiomycetes were retrieved from GenBank. The related sequences were obtained from a BLAST search and from recently published data (Ekanayaka et al., 2019). Individual datasets for LSU and ITS genes were aligned using the default settings of MAFFT V.7.036^1^ (Katoh et al., 2018) and improved manually where necessary using Bioedit. Aligned gene regions were concatenated using Bioedit v.7.2 (Hall, 1999) and analyzed.

Initial alignment of LSU region included 7163 base pairs and ITS region included 6619 base pairs. In the phylogenetic analysis, LSU and ITS regions consisted of ambiguously aligned regions. Hence, manual alignment was performed where necessary and some unambiguous regions were removed from the analysis. The removed regions of LSU data set are 0–2658, 2738–2888, 2897–2955, 3037–3220, 3285–3338, 3421–3464, 3723–3730, 4029–4103, 4129–4562, 4581–7163. The excluded regions of the ITS data set are 0–146, 149–173, 181–2164, 2199–2234, 2253–2307, 2714–2923, 2936–2971, 2986–3052, 3065–3113, 3132–3270, 3331–6619. In the final alignment, LSU and ITS regions consist of 898 and 587 bp, respectively.

Phylogenetic constructions of combined gene trees were performed using maximum likelihood (ML), maximum parsimony (MP) and bayesian inference (BI) criteria. Maximum likelihood trees were generated using the RAxML-HPC2 on XSEDE (8.2.8) (Stamatakis et al., 2008; Stamatakis, 2014) in the CIPRES Science Gateway platform (Miller et al., 2010) using the GTR+I+G model of evolution. The robustness of the most parsimonious tree was estimated based on 1000 bootstrap replications.

Maximum parsimony analysis was carried out in PAUP (Phylogenetic Analysis Using Parsimony) v. 4.0b10 (Swofford, 2002) using the heuristic search option, random stepwise addition, and 1000 replicates, with maxtrees set at 1000. Descriptive tree statistics for parsimony such as Tree Length [TL], Consistency Index [CI], Retention Index [RI], Relative Consistency Index [RC] and Homoplasy Index [HI] were calculated for trees generated under different optimality criteria. The Kishino Hasegawa tests (Kishino and Hasegawa, 1989) were performed to determine whether the trees inferred under different optimality criteria were different.

Evolutionary models for phylogenetic analyses were selected independently for each locus using MrModeltest v. 3.7 (Nylander, 2004) under the Akaike Information Criterion (AIC) implemented in both PAUP v. 4.0b10 and MrBayes v. 3.

Bayesian inference analysis was conducted with MrBayes v. 3.1.2 (Huelsenbeck and Ronquist, 2001) to evaluate posterior probabilities (BYPP) (Rannala and Yang, 1996; Zhaxybayeva and Gogarten, 2002) by Markov Chain Monte Carlo sampling (BMCMC). Kimura 2-parameter model coupled with discrete gamma distribution with a proportion of invariant site (TrN+I+G) was applied for LSU gene region and symmetrical model with discrete gamma distribution coupled with a proportion of invariant sites (TIM2ef+I+G) was applied for ITS gene region. Two parallel runs were conducted, using the default settings, but with the following adjustments: Four simultaneous Markov chains were run for 2,000,000 generations and trees were sampled every 100^th^ generation. The distribution of log-likelihood scores indicated the stationary phase for each search and were used to decide if extra runs were required to achieve convergence, using Tracer v. 1.6 (Rambaut et al., 2014). The first 20% of the generated trees represented the burn-in phase and were discarded. The remaining trees were used to calculate posterior probabilities of the majority rule consensus tree.

Phylograms were visualized with FigTree v1.4.0 (Rambaut, 2012) and edited in Microsoft Power Point (2016) and Adobe Illustrator CS5 (Version 15.0.0, Adobe, San Jose, CA, United States). The finalized alignment and the tree were deposited in TreeBASE, submission ID: 24892^2^.

## Results

### Phylogenetic Analyses

Phylogenetic trees obtained from LSU and ITS single gene analyses as well as the combined gene analyses share similar overall topologies at the generic level and are in agreement with previous studies (Johnston et al., 2014; Crous et al., 2015; Ekanayaka et al., 2019). The concatenated LSU and ITS dataset consisted of 58 taxa.

**FIGURE 1:**
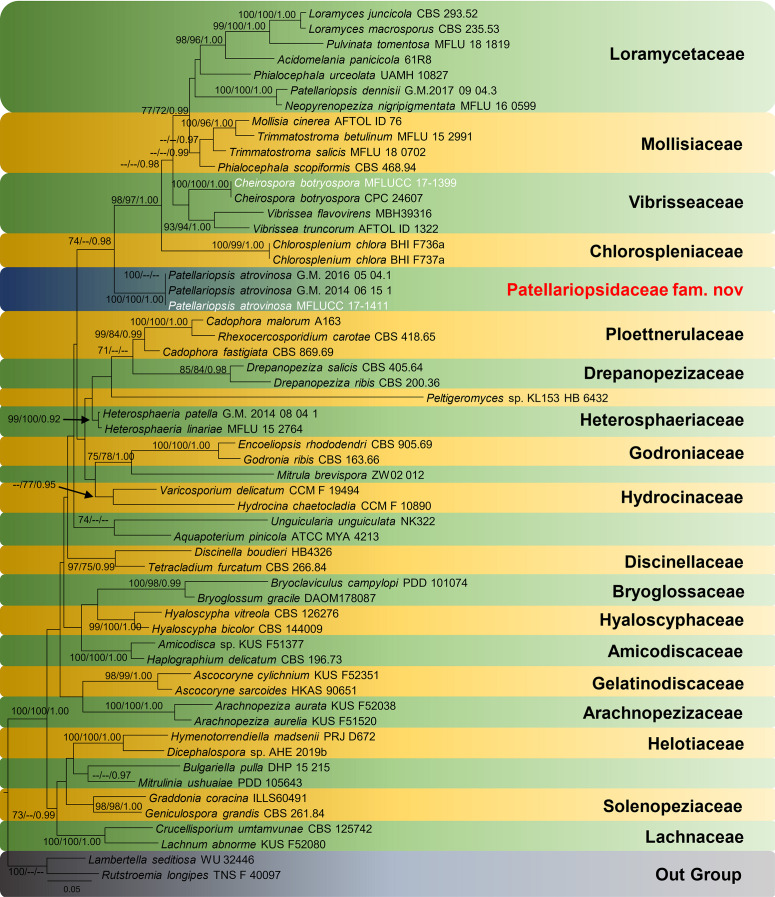
RAxML tree based on a combined dataset of LSU and ITS partial sequence data. Bootstrap support values for maximum likelihood equal to or higher than 70%, maximum parsimony equal to or higher than 70%, and Bayesian posterior probabilities equal to or greater than 0.90 are displayed on the nodes, respectively. Newly generated sequences are indicated in white. The tree is rooted to *Lambertella seditiosa* and *Rutstroemia longipes.*

The RAxML analysis of the LSU dataset yielded a best scoring tree (Figure 1) with a final ML optimization likelihood value of −16138.064322. The matrix had 758 distinct alignment patterns, with 19.46% of undetermined characters or gaps. Parameters for the GAMMA+P-Invar model of the LSU and ITS were as follows: Estimated base frequencies; A = 0.247456, C = 0.223115, G = 0.280101, T = 0.249328; substitution rates AC = 1.847670, AG = 2.484717, AT = 1.566081, CG = 1.283952, CT = 5.746161, GT = 1.000000; proportion of invariable sites I = 0.458978; gamma distribution shape parameter α = 0.633036. The maximum parsimony dataset consisted of 1490 characters, of which 860 were constant, 475 parsimony-informative and 155 parsimony-uninformative. The parsimony analysis of the data matrix resulted in four equally most parsimonious trees with a length of 948 steps (CI = 0.347, RI = 0.556, RC = 0.193, HI = 0.653) in the best tree.

### Taxonomy

In this section, Patellariopsidaceae Karun., Camporesi & K.D. Hyde, fam. nov. and the new record of *Cheirospora botryospora* are described and illustrated. Helotiales includes several families with sporodochial asexual morphs *viz*. Gelatinodiscaceae, Helotiaceae and Mollisiaceae. A morphological comparison among members of the families in Helotiales is given in Tables 2, 3.

**TABLE 2:** Comparison of major asexual morph characteristics of families in order Helotiales.

**Family**	**Hyphomycetous conidiomata**	**Conidiophore**	**Conidiogenous cell**	**Conidia**
Amicodiscaceae (Ekanayaka et al., 2019)	Hyphomycetous/stromatic	Hyaline to cinnamon-colored glistening slimy heads, straight or flexuous, dark brown and thick-walled except at the apex	Terminal, cylindrical, sympodially proliferate	Cylindrical to cylindric-ellipsoidal, hyaline, aseptate, thin-smooth walled.
Discinellaceae (Ekanayaka et al., 2019)	Hyphomycetous conidiomata		Holoblastic	Mostly hyaline, sometimes branched, filiform, globose, or fusoid some form dimorphic conidia
Drepanopezizaceae (Yoshikawa and Yokoyama, 1992; König et al., 2017)	Hyphomycetous/acervulus		Holoblastic	Sometimes two types. Macroconidia- ellipsoid to fusoid, slight curved. Microconidia- ellipsoid to bacilliform
Gelatinodiscaceae (Seaver, 1938; Johnston et al., 2010)	Sporodochial			Aseptate, hyaline and subglobose
Helotiaceae (Peláez et al., 2011; Jaklitsch et al., 2016)	Hyphomycetous, sporodochial or synnematal		Macroconidia – holoblastic/Microconidia – phialidic	Macroconidia – hyaline, filiform or staurosporous, dark brown, in chains, bulbils or solitary on conidiophores and 3–5-septate. Microconidia rarely pigmented, multicellular and appendaged
Heterosphaeriaceae (Leuchtmann, 1987)	Synanamorphic, hyphomycetous acervulus and ceolomycetous			
Hyaloscyphaceae (Jaklitsch et al., 2016)	hyphomycetous	sporodochial	Phialidic	Aseptate, hyaline or brown, branched and muriform or in chains
Hydrocinaceae (Ekanayaka et al., 2019)	Hyphomycetous	Long, hyaline, simple or branched, filiform	Proliferate, sympodial.	Filiform, branched, sometimes septate and fragment into microconidia.
Loramycetaceae (Digby and Goos, 1987; Walsh et al., 2014)	anguillospora-like	Conidiophores are simple or occasionally branch. Conidiogenous cells are hyaline and straight. Conidia are globose, sub-ellipsoid or sigmoid and hyaline	Conidiogenous cells are hyaline and straight. Conidia are globose, sub-ellipsoid or sigmoid and hyaline	Conidia are globose, sub-ellipsoid or sigmoid and hyaline
Mollisiaceae (Sutton and Ganapathi, 1978; Butin et al., 1996; Grünig et al., 2002)	Sporodochial	Hyaline to brown		Unicellular, ellipsoid or phragmosporous, hyaline or brown and also in chains
Patellariopsidaceae	Sporodochium	Cylindrical, straight or slightly curved, branched over the conidiophore, septate, hyaline, expanding toward the apices, smooth	Holoblastic, polyblastic, cylindrical, integrated, hyaline, smooth.	Sphaerical, acropetal, branched chains. globose to cylindrical mass of small, thick-walled, dark brown, septate, eguttulate, smooth, cheiroid, conidium-complex
*Phialocephala urceolata* clade Wang, 2009	Hyphomycetous	Hyaline to darkly pigmented, septate and mononematous	Phialidic and conidiogenous cells are flask to urn-shaped and each with a prominent cylindrical and hyaline collarette	Globose, pedicellate and single or adhering in small clusters at the phialide apex
Ploettnerulaceae (Marvanová and Bärlocher, 2001; Goodwin, 2002; Gönczöl and Révay, 2003; Gramaje et al., 2011; Gonçalves et al., 2012; King et al., 2013; Travadon et al., 2015; Duarte et al., 2016; Walsh et al., 2018)	hyphomycetous or coelomycetous	Hyaline to brown	Phialidic	Ellipsoid to rod-shaped or filiform with pointed apices and 0–1-septate
Solenopeziaceae (Ekanayaka et al., 2019)	Conidiomata hyphomycetous	Simple, sparsely branched or absent	Cylindrical to subclavate, sometimes apically slightly swollen	Hyaline or black, septate, branched, lunate, sometimes formed in a chain and becoming tortuous and appearing as terminal dictyospores, rarely appendaged
Vibrisseaceae (Iturriaga and Israel, 1985; Goh and Hyde, 1998; Goh et al., 1998; Kirschner and Oberwinkler, 2001; Shenoy et al., 2010; Hernández-Restrepo et al., 2012, 2017; Legon, 2012; Crous et al., 2015)	hyphomycetous, phialidic and acervulus	Straight, cylindrical, hyaline and sometimes branched	Holoblastic or polytretic	Ellipsoid or irregular in shape and unicellular or up to 7–septate

**TABLE 3:** Comparison of major sexual morph characteristics of families in order Helotiales based on Ekanayaka et al. (2019).

**Family**	**Ascomata**	**Excipulum Peridium**	**Paraphyses**	**Asci**	**Ascospores**
Amicodiscaceae	Apothecial, cupulate, sessile or sub-stipitate, margins covered by hairs	Ectal excipulum textura angularis or textura prismatica cells, medullary excipulum loosely arranged hyphae	Filiform, cylindrical, septate, simple	8-spored, amyloid, sometimes arising from croziers	Ellipsoid to fusoid, aseptate, guttulate, lemon-yellow pigmented
Aquapoterium Unguicularia clade	Apothecial, cupulate receptacle, sessile or tipitate, sometimes margins covered with short cylindrical hairs	Ectal excipulum textura prismatica cells or a single layer of parallel hyphae with enlarged, globose apices, medullary excipulum reduced or composed of loosely arranged hyphae	Filiform, hyaline, obtuse to lavate at apex, septate, smooth-walled, simple or branched	8-spored, amyloid or non-amyloid, cylindric-clavate	Ellipsoid to clavate cylindric, hyaline, smooth-walled, 0–1-septate, surrounded by a gelatinous sheath
Arachnopezizaceae	Apothecial, covered by hairs	Ectal excipulum textura angularis to prismatica cells, medullary excipulum textura prismatica to textura oblita cells	Cylindrical, hyaline	8-spored, cylindric clavate, amyloid, arising from croziers	Ellipsoid to fusoid, 0–7-septate
Bryoglossaceae	Apothecial, clavate to apitate or cupulate to turbinate, long stipitate, gelatinous	Ectal excipulum textura porrecta cells, medullary excipulum textura intricata cells	Filiform, swollen at the apex	8-spored, amyloid or non-amyloid, arising from croziers	Ellipsoid to fusoid, straight, aseptate, guttulate
*Bulgariella* clade	Apothecial or rarely cleistothecial, cupulate, discoid, turbinate or capitate, sessile or stipitate, margins and flanks are covered with hairs	Ectal excipulum is composed textura angularis, textura prismatica or textura oblita cells, medullary excipulum is composed of cells of textura intricata or textura oblita cells	Filiform, lanceolate or cylindrical	8-spored, cylindric clavate, amyloid or non-amyloid, sometimes arising from croziers	Globose, ellipsoid to filiform, septate or aseptate, hyaline or brownish, guttulate
Chlorospleniaceae	Apothecial, cupulate or discoid, sessile or substipitate	Ectal excipulum textura angularis cells, medullary excipulum textura intricata cells	Filiform, septate	8-spored, cylindric clavate, amyloid	Ellipsoid to fusoid, hyaline and smooth walled
*Colipila* clade	Apothecial cupulate, covered by long cylindrical hairs	Ectal excipulum and medullary excipulum textura prismatica cells	Dimorphic, sub cylindrical and not exceed the length of asci, or broadly lanceolate and exceed the length of asci	8-spored, cylindric– clavate, amyloid, arising from croziers	Ellipsoid to fusoid
Discinellaceae	Apothecial, discoid to cupulate, circular, gelatinous, sometimes covered with hairs	Ectal excipulum textura prismatica or textura porrecta cells, medullary excipulum textura intricata to prismatica cells	Filiform, branched at the apices	8-spored, cylindrical, amyloid or non amyloid, sometimes arising from croziers	Ellipsoid, aseptate, hyaline, without sheath
Drepanopezizaceae	Apothecial, cupulate, sessile, mostly immersed	A thin layer of textura angularis cells,	Apically slightly swollen, straight	4–8- spored, non amyloid	Ellipsoid to fusoid, 0–2-septate
Gelatinodiscaceae	Apothecial, cupulate or discoid, some are tremelloid, form cerebriform masses which each lobule contains a turbinate apothecium	Ectal excipulum textura prismatica to textura angularis to globulosa cells, medullary excipulum textura oblita to textura porrecta or textura intricata cells	Filiform, cylindrical, apically swollen, guttulate	8-spored, amyloid, arising from croziers	Ellipsoid to fusoid, hyaline, yellowish or brownish, smooth, with a gelatinous sheath, guttulate, 0–5-septate
Godroniaceae	Apothecial, urceolate, discoid or cupulate, mostly stromatic, erumpent, sometimes covered with hairs	Ectal excipulum textura prismatica to angularis cells, medullary excipulum textura epidermoidea, prismatica to porrecta cells	Filiform or lanceolate, simple or branched, sometimes slightly swollen at the apex	8-spored, cylindric clavate, amyloid or non-amyloid	Fusoid, hyaline, septate, guttulate
Helotiaceae	Apothecial, cupulate, discoid, capitate to clavate, turbinate or globose, sessile or tipitate, margins and flanks smooth or covered with hairs	Ectal excipulum textura prismatica, intricata, globulosa-angularis, or toblita cells, medullary excipulum textura intricata or porrecta cells	Cylindrical, septate or aseptate, hyaline to yellowish, guttulate	4–8-spored, cylindric-clavate, amyloid or non amyloid, sometimes arising from croziers	Ellipsoid, fusoid or filiform, 1–3-septate, rarely ornamented
Heterosphaeriaceae	Apothecial, discoid, black, sessile, erumpent, gelatinous	Ectal excipulum textura angularis cells, medullary excipulum textura porrecta cells	Clavate contains many guttules	8-spored, amyloid, arising from croziers	Aseptate, ellipsoid to fusoid, without gel sheath
Hyaloscyphaceae	Apothecial, cupulate or discoid, sessile or substipitate, sometimes covered with hairs	Ectal excipulum textura globulosa cells, medullary excipulum textura porrecta, intricata to oblita cells	Filiform, septate, branched, slightly swollen at the apices	8-spored, cylindric clavate, amyloid, arising from croziers	Ellipsoid to fusoid, aseptate or septate, hyaline
Hydrocinaceae	Apothecial, cupulate, sessile or substipitate	Ectal excipulum textura globulosa cells, medullary excipulum textura porrecta, intricata or oblita cells	Filiform, septate, branched, slightly swollen at the apices	8-spored, cylindric clavate, amyloid, arising from croziers	Ellipsoid to fusoid, aseptate or septate, hyaline
Lachnaceae	Apothecial, cupulate or discoid, sessile or stipitate, margins and flanks are covered with hairs	Ectal excipulum textura angularis, prismatica or oblita cells, medullary excipulum textura intricata or textura oblita cells	Filiform, lanceolate or rarely cylindrical	8-spored, cylindric clavate, amyloid or non-amyloid, sometimes arising from croziers	Globose, ellipsoid to filiform or allantoid, septate or aseptate, hyaline, guttulate
Loramycetaceae	Apothecial or perithecial, apothecia cupulate or pulvinate, perithecia sub-globose	Ectal excipulum textura prismatica, angularis or globulosa cells, medullary excipulum textura prismatica cells	Filiform, septate, unbranched, sometimes apically swollen and pigmented	8-spored, cylindric clavate, amyloid or non-amyloid	Fusiform, septate, sometimes with terminal appendages and gel sheath
Mitrulaceae	Apothecial, clavate, stipitate	Ectal excipulum textura porrecta cells, medullary excipulum textura intricata cells	Filiform, cylindrical, with yellow carotenoid droplets	8-spored, cylindric clavate, arising from croziers	Fusoid to ellipsoid, straight or curved
Mollisiaceae	Apothecial, discoid covered by hairs,	Ectal excipulum textura globulosa to angularis cells, medullary excipulum textura prismatica cells	Cylindrical or lanceolate, apically swollen, guttulate	8-spored, amyloid, cylindric clavate, mostly arising from croziers	Ellipsoid to long filiform, 0–7-septate, guttulate
Patellariopsidaceae	Apothecial, discoid, sessile	Ectal excipulum textura globulosa to angularis cells, medullary excipulum interwoven refractive hyphae	filiform, branched and pigmented at the apices	8-spored, cylindric clavate, amyloid	Ellipsoid to fusoid, hyaline, 3–7-septate
Peltigeromyces clade	Apothecial, cartilaginous, thin, with a large variety of lobes	Records are not available for micro morphological characters			
Phialocephala urceolata clade	Sexual morphs are not recorded				
Ploettnerulaceae	Apothecial, cupulate, discoid or urn-shaped, sessile or sub stipitate, sometimes covered with pigmented hairs	Ectal excipulum textura globulosa to angularis cells, medullary excipulum textura prismatica cells	Filiform, cylindrical or lanceolate, guttulate	8-spored, conical apex, amyloid	Ellipsoid to long filiform, 0–3-septate, guttulate
Solenopeziaceae	Apothecial cupulate, discoid or pulvinate, sessile or stipitate, sometimes covered with hyaline, whitish, yellow or brown, non-bristle like hairs	Ectal excipulum textura angularis, textura prismatica or textura oblita cells, medullary excipulum textura intricata or textura oblita cells	Filiform, lanceolate or cylindrical	8-spored, cylindric clavate, amyloid or non-amyloid, sometimes arising from croziers	Globose, ellipsoid to fusiform, septate or aseptate, guttulate
Vibrisseaceae	Apothecial, cupulate or clavate, sessile to stipitate	Ectal excipulum textura angularis to globulosa cells, medullary excipulum reduced or textura oblita cells	Filiform, apically slightly swollen, sometimes branched	8-spored, cylindric clavate, long stipitate, sometimes amyloid, arising from croziers	

### Patellariopsidaceae Karun., Camporesi and K.D. Hyde, Fam. Nov.

Index Fungorum number: IF556719, Facesoffungi number: FoF06573

**Sexual Morph**: *Ascomata* apothecial, discoid, sessile or stipitate. Ectal excipulum composed of cells of textura globulosa to angularis cells. Medullary excipulum composed of interwoven refractive hyphae. *Paraphyses* filiform branched and pigmented at the apices. *Asci* 8-spored, cylindric-clavate, amyloid. *Ascospores* ellipsoid to fusoid, hyaline, 3–7-septate. Asexual morphs: *Saprobic* on dead branch of *Corylus avellana* (Betulaceae). **Asexual morph:** *Sporodochium*, sub-epidermal or sub-peridermal, solitary. *Conidiophores* cylindrical, straight or slightly curved, branched over the conidiophore, septate, hyaline, expanding toward the apices, smooth. *Conidiogenous cells* holoblastic, polyblastic, cylindrical, integrated, hyaline, and smooth. *Conidia*, sphaerical, proliferating with several, short, lateral, acropetal, branched chains. Primary branches in turn develop secondary branches, which eventually form a globose to cylindrical mass of small, thick-walled, dark brown, septate, eguttulate, smooth, cheiroid, conidium-complex.

#### Notes

Patellariopsidaceae forms a well-supported (ML 74/BYPP 0.98) clade sister to Chlorospleniaceae, Loramycetaceae, Mollisiaceae and Vibrisseaceae. In Index Fungorum, *Patellariopsis* is included in Dermateaceae, but Wijayawardene et al. (2017) placed *Patellariopsis* in Helotiales genera *incertae sedis* based on morphology. Furthermore, in Ekanayaka et al. (2019), this clade was denoted as separate taxa based on phylogenetic analyses. Hence, we introduce this clade as a new family based on morphology and phylogeny.

#### Type Genus

*Patellariopsis* Dennis, Kew Bull. 19(1): 114 (1964)

### *Patellariopsis* Dennis, Kew Bull. 19(1): 114 (1964)

Index Fungorum number: IF556217, Faces of Fungi number: FoF06575

The genus classified under Helotiales genera *incertae sedis*, Leotiomycetes (Wijayawardene et al., 2018). The type species is *Patellariopsis clavispora* (Berk. & Broome) Dennis. Five species are recorded in Index Fungorum (2019), *P. atrovinosa* (A. Bloxam ex Curr.) Dennis, *P. carnea* G.W. Beaton, *P. clavispora* (Berk. & Broome) Dennis, *P. dennisii* (E. Müll. & Hütter) Schläpf.-Bernh., and *P. indica* A. Pande. We were unable to find any reported described asexual morphs of *Patellariopsis* in the literature.

### *Patellariopsis atrovinosa* (A. Bloxam ex Curr.) Dennis, Kew Bull. 29(1): 167 (1974)

**FIGURE 2:**
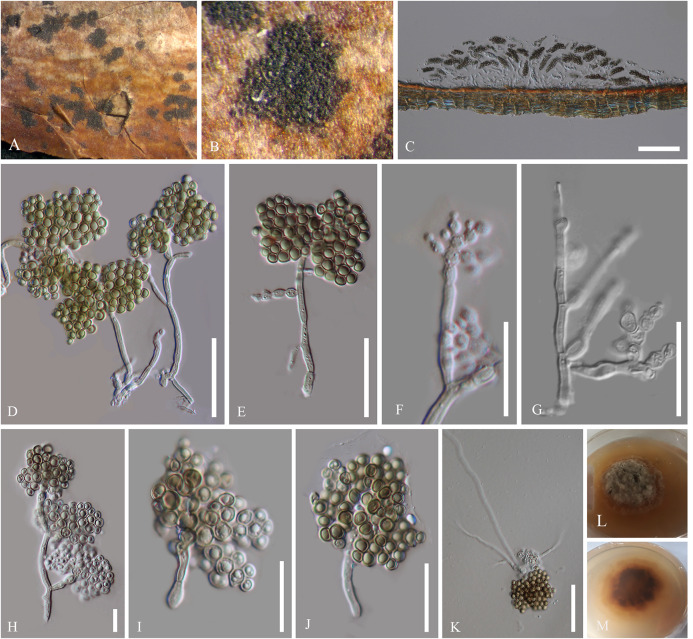
Asexual morph of *Patellariopsis atrovinosa* (MFLU 16-2950). **(A,B)** Appearance of sporodochium on host substrate. **(C)** Longitudinal section of sporodochium. **(D)** Conidiophore attached to the host. **(E–G)** Various stages of conidiogenesis. **(H–J)** Conidia. **(K)** Germinated conidium. **(L,M)** Culture characteristics on PDA (**L** = from above, **M** = from below). Scale bars: **C** = 50 μm; **D,E** = 20 μm; **F–K** = 10 μm.

Index Fungorum number: IF 319233, Facesoffungi number: FoF06574 Figure 2

*Saprobic* on dead branch of *Corylus avellana* (Betulaceae). **Sexual morph:** Refer to Dennis (1974). **Asexual morph:** *Sporodochium* 33–37 μm high, 278–355 μm diam. ($\bar{x}$ = 35 × 324 μm, *n* = 5), sub-epidermal or sub-peridermal, solitary. *Conidiophores* 41–78 × 1–1.5 μm ($\bar{x}$ = 58 × 1.3 μm,

*n* = 20) cylindrical, straight or slightly curved, branched over the conidiophore, septate, hyaline, expanding toward the apices, smooth. *Conidiogenous cells* 1.5–2 × 1–1.6 μm ($\bar{x}$ = 1.8 × 1.4 μm, *n* = 20) holoblastic, polyblastic, cylindrical, integrated, hyaline, smooth. *Conidia* 1.5–2.7 × 1.5–2.5 μm ($\bar{x}$ = 2 × 2 μm, *n* = 40), sphaerical, proliferating with several, short, lateral, acropetal, branched chains. Primary branches in turn develop secondary branches, which eventually form a globose to cylindrical mass of small, thick-walled, dark brown, septate, eguttulate, smooth, cheiroid, conidium-complex.

Colonies growing on PDA becoming 2 cm within 10 days at 16°C, circular, flat, cottony, irregular margin, with less aerial mycelium, olivaceous green to gray from above and dark brown from below.

#### Material Examined

ITALY, Forlì-Cesena [FC], Fiumicello di Premilcuore, dead aerial branch of *Corylus avellana* L. (Betulaceae), 5 September 2015, E. Camporesi, IT 3178 (MFLU 16-2950), living cultures, MFLUCC 17-1411.

### *Cheirospora* Moug. & Fr., in Fries, Syst. Orb. Veg. (Lundae) 1: 365 (1825)

Index Fungorum number: IF 7614, Faces of Fungi number: FoF06593

The genus is in Helotiales genera *incertae sedis*, Leotiomycetes (Wijayawardene et al., 2018). Ekanayaka et al. (2019) placed this genus under Vibrisseaceae. The type species is *C. botryospora* (Mont.) Berk. & Broome. There are four species in Index Fungorum (2019), *C. alni* Shabunin., *C. betulina* (P. Karst.) Kuntze., *C. botryospora* (Mont.) Berk. & Broome and *C. oblonga* (Fuckel) Kuntze.

***Cheirospora botryospora*** (Mont.) Berk. & Broome, Ann. Mag. nat. Hist., Ser. 2 5: 455 (1850)

**FIGURE 3:**
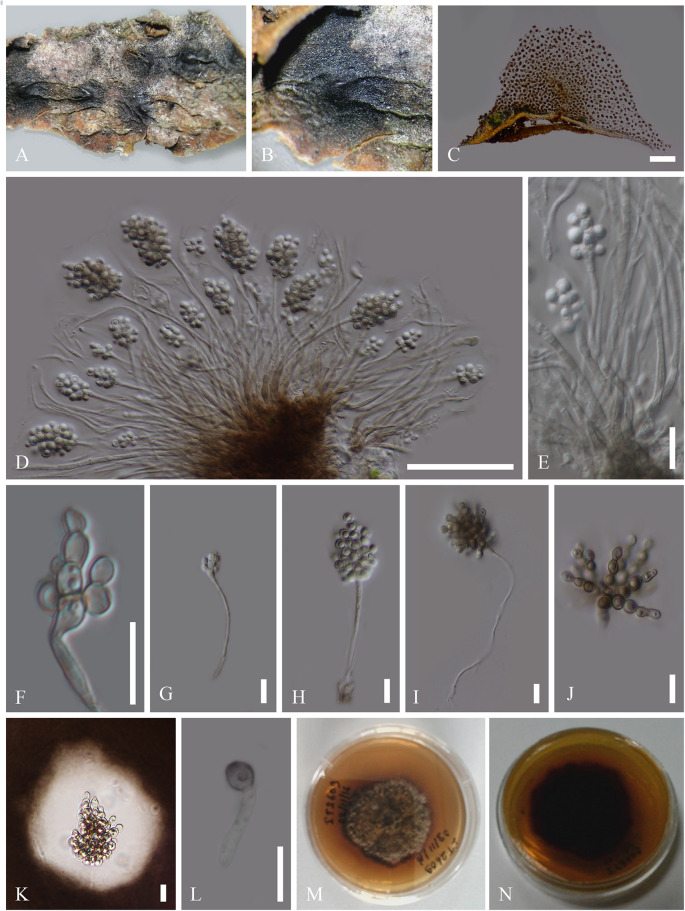
Asexual morph of *C. botryospora* (MFLU 15-2612). **(A,B)** Appearance of sporodochium on host substrate. **(C)** Longitudinal section of sporodochium. **(D,E)** Conidia attached to the conidiophores. **(F–J)** Conidia. **(K)** Conidia surrounded by mucilaginous sheath, stained with Indian ink. **(L)** Germinated conidium. **(M,N)** Culture characteristics on PDA (**M** = From above, **N** = From below). Scale bars: **C** = 20 μm; **D,E** = 50 μm; **F–J** = 10 μm.

Index Fungorum number: IF 294800, Facesoffungi number: FoF06594 (Figure 3)

*Saprobic* on dead branches of *Fagus sylvatica* L. **Sexual morph***:* unidentified. **Asexual morph:** *Sporodochium* 1850–1854 μm high, 3728–3732 μm diam. ($\bar{x}$ = 1852 × 1730 μm, *n* = 5), sub-epidermal or sub-peridermal, solitary. *Conidiophores* 171–225 × 3–4 μm ($\bar{x}$ = 198 × 3.5 μm, *n* = 20) cylindrical, straight or slightly curved, branched only at the base, septate, hyaline, expanding toward the apices, smooth. *Conidiogenous cells* 8–7 × 10–11 μm ($\bar{x}$ = 7.5 × 10.5 μm, *n* = 20) holoblastic, polyblastic, cylindrical, integrated, hyaline, smooth. *Conidia* 10–11 × 9–11 μm ($\bar{x}$ = 10.5 × 10 μm, *n* = 40), sphaerical, proliferating with several, short, lateral, acropetal, branched chains. Primary branches in turn develop secondary branches which eventually form a globose to cylindrical mass of small, thick-walled, dark brown, septate, eguttulate, smooth, cheiroid, conidium-complex, enclosed in a gelatinous sheath.

Colonies growing on PDA to 2 cm diam. within 10 days at 16°C, circular, flat, cottony, irregular margin, with less aerial mycelium, olivaceous green to gray from above and dark brown from below.

#### Material Examined

ITALY, Province of Arezzo [AR], Passo la Calla - Stia, dead aerial branch of *Fagus sylvatica* (Fagaceae), 5 September 2015, E. Camporesi, IT 2609 (MFLU 15-2612), ex-type living culture, MFLUCC 17-1399.

## Discussion

The highly divergent morphological, ecological and biological characteristics of Helotiales makes it a focus for taxonomic studies in the Leotiomycetes, as it is one of the most problematic groups for traditional classification and molecular phylogeny (Wang et al., 2006a). It is a poorly studied order, within which about 19–27% of the genera have an uncertain position at the family level (Baral, 2016). Hence, the taxa in Helotiales have already been subjected to several nomenclatural reinterpretations (Wang et al., 2006a). Lantz et al. (2011) revealed that some genera related to members in Helotiales were traditionally placed in Rhytismatales.

*Patellariopsidaceae* is established herein based on morphological and phylogenetic support. Comparisons of major sexual and asexual morph characteristics of families in Helotiales are provided in Tables 2 and 3. The asexual morph characteristics of this family are unique in having sporodichium with cheiroid conidium complex. The cheiroid conidium complexes are also present in *C. botryospora* in Vibrisseaceae. Patellariopsidaceae differs from Vibrisseaceae, in having highly branched conidiophores and thicker conidia complexes. Further, the sexual morph of the Patellariopsidaceae shows unique characteristics by having a sessile discoid apothecium, paraphyses with filiform, branched and pigmented apices, cylindric-clavate, amyloid asci and ellipsoid to hyaline septate ascospores. Patellariopsidaceae was further supported by phylogeny. Hence, herein we establish the Patellariopsidaceae under Helotiales.

Most of the *Patellariopsis* species were recorded from the United Kingdom with few exceptions (Beaton and Weste, 1978; Farr and Rossman, 2020). *Patellariopsis atrovinosa* on *Prunus laurocerasus* was also reported from the United Kingdom. In our study, we report the asexual morph of *P. atrovinosa* on *Corylus avellana* from Italy. *Patellariopsis carnea* on dead grass twigs was reported from Australia (Beaton and Weste, 1978). *P. clavispora* shows a wide host range, which includes *Acer* sp., *Corylus* sp., *Crataegus* sp., *Fagus* sp., *Fraxinus* sp., *Ligustrum* sp., *Prunus* sp., *Quercus* sp. and *Symphoricarpos* sp. from the United Kingdom (Dennis, 1978, 1986) and *Mangifera indica* from Pakistan (Ahmad, 1978).

Apart from ribosomal RNA sequence data, the use of protein-coding gene phylogenies involving helotialean fungi are slowly emerging (Wang et al., 2006b; Johnston et al., 2014). Most contemporary results suggest that the Helotiales and currently delimited families are not monophyletic and that the highly conserved small subunit (SSU) rRNA gene is not informative enough to resolve these lineages with confidence (Gernandt et al., 2001).

In our phylogenetic analyses, all the *Patellariopsis* strains available in the GenBank were included. Among them, the phylogenetic placement of *P. dennisii* (G.M. 2017 09 04.3) is ambiguous. No morphological descriptions are available in the literature for comparison (Ekanayaka et al., 2019; Vu et al., 2019) and the topology obtained in this study is similar to the topology obtained by Ekanayaka et al. (2019). Hence, we suggest the need for having more data to clarify the position of *P. dennisii* (G.M. 2017-09-04.3). The blast results for the *Patellariopsis dennisii* (CBS 174.66) strain include several other Ascomycetous fungi. Therefore, *Patellariopsis dennisii* (CBS 174.66) was excluded in our dataset after the preliminary phylogenetic analyses.

Genealogical Concordance Phylogenetic Species Recognition (GCPSR) analysis using multi-gene concatenated sequences is used to determine the recombination level within phylogenetically closely related species. Under this study three *P. atrovinosa* strains MFLUCC 17-1411, G.M. 2016-05-04.1, G.M. 2014-06-15.1, and *P. dennisii* G.M. 2017 09 04.3 were subjected to the GCPSR analysis. The analysis failed due to the lack of the informative characters in the highly similar sequences of *P. atrovinosa* strains MFLUCC 17-1411, G.M. 2016-05-04.1 and G.M. 2014-06-15.1.

Based on phylogenetic analyses, our strain MFLUCC 17-1411 forms a well-supported (ML 100/ MP 100/BYPP 1.00) clade with specimens G.M. 2016-05-04.1 and G.M. 2014-06-15.1 (both *P. atrovinosa*). The phylogenetic relatedness is supported by the 100% similarity between sequences. The asexual stage of *Patellariopsis* is not recorded in literature. Hence, no morphological comparison can be done between the strains MFLUCC 17-1411 and *P. atrovinosa.* However, Meyer & Carrières (Meyer and Carrières, 2007) have described an asexual hyphomycetous *Periconia-*like association for the sexual morph *P. atrovinosa.* Nevertheless, no scientific evidence was provided to confirm this association. Hence, we justify MFLUCC 17-1411 belongs to the *P. atrovinosa.*

*Cheirospora botryospora* MFLUCC 17-1399 forms a well-supported clade with (ML 100/MP 100/BYPP 1.00) *C. botryospora* CPC 24607 and this was further supported by morphology. *C. botryospora* CPC 24607 was isolated from *Fagus sylvatica* in Germany. Danti et al. (2002) identified an endophytic *Cheirospora* sp. on *Fagus sylvatica* from Italy. However, they were unable to identify it to the species level. Therefore, in this study, based on morphology and phylogeny the first report of *C. botryospora* on *F. sylvatica* from Italy is provided (Farr and Rossman, 2020).

In this study, the Patellariopsidaceae fam. nov. is introduced with an asexual morph. Furthermore, a new host record for the *C. botryospora* (Vibrisseaceae) and updated phylogenetic tree for Helotiales are provided.

## Data Availability Statement

The datasets analyzed in this manuscript are not publicly available. Requests to access the datasets should be directed to anumandrack@yahoo.com.

## Author Contributions

AK and KH designed the study. AK performed the morphological study and phylogenetic the data analyses with the help of DP, AE, KC, and RJ. DP did the primer design. SL and SK provided the grant. AK wrote the manuscript. RJ, DP, IG, KC, AE, SK, SL, RC, and KH reviewed and edited the manuscript. All authors reviewed and approved the final manuscript.

## Conflict of Interest

The authors declare that the research was conducted in the absence of any commercial or financial relationships that could be construed as a potential conflict of interest. The reviewer RJ declared a past co-authorship with the authors AK, DP, AE, RJ, KC, IG, RC, EC, KH, SL, SK to the handling editor.

## Footnotes

1 http://mafft.cbrc.jp/alignment/server/

2 http://www.treebase.org
